# Mechanisms of Scarless Repair at Time of Menstruation: Insights From Mouse Models

**DOI:** 10.3389/frph.2021.801843

**Published:** 2022-01-06

**Authors:** Phoebe M. Kirkwood, Isaac W. Shaw, Philippa T. K. Saunders

**Affiliations:** Centre for Inflammation Research, The University of Edinburgh, Edinburgh, United Kingdom

**Keywords:** hypoxia, endometrium, mesenchyme to epithelial transition (MET), inflammation, cytokine, angiogenesis, scarless

## Abstract

The human endometrium is a remarkable tissue which may experience up to 400 cycles of hormone-driven proliferation, differentiation and breakdown during a woman's reproductive lifetime. During menstruation, when the luminal portion of tissue breaks down, it resembles a bloody wound with piecemeal shedding, exposure of underlying stroma and a strong inflammatory reaction. In the absence of pathology within a few days the integrity of the tissue is restored without formation of a scar and the endometrium is able to respond appropriately to subsequent endocrine signals in preparation for establishment of pregnancy if fertilization occurs. Understanding mechanisms regulating scarless repair of the endometrium is important both for design of therapies which can treat conditions where this is aberrant (heavy menstrual bleeding, fibroids, endometriosis, Asherman's syndrome) as well as to provide new information that might allow us to reduce fibrosis and scar formation in other tissues. Menstruation only occurs naturally in species that exhibit spontaneous stromal cell decidualization during the fertile cycle such as primates (including women) and the Spiny mouse. To take advantage of genetic models and detailed time course analysis, mouse models of endometrial shedding/repair involving hormonal manipulation, artificial induction of decidualization and hormone withdrawal have been developed and refined. These models are useful in modeling dynamic changes across the time course of repair and have recapitulated key features of endometrial repair in women including local hypoxia and immune cell recruitment. In this review we will consider the evidence that scarless repair of endometrial tissue involves changes in stromal cell function including mesenchyme to epithelial transition, epithelial cell proliferation and multiple populations of immune cells. Processes contributing to endometrial fibrosis (Asherman's syndrome) as well as scarless repair of other tissues including skin and oral mucosa are compared to that of menstrual repair.

## Introduction

The endometrium is unusual amongst adult tissue in that it exhibits an unparalleled capacity for rapid scar-free repair, which occurs at the end of each non-fertile cycle during the phase known as menstruation. Menstruation is the culmination of vascular, cellular and inflammatory changes which leaves the luminal surface in a “wounded” state (1). In order to limit blood loss and regain tissue function for the subsequent cycle, rapid re-epithelialisation and structural re-organization is required and occurs without the accumulation of any functional damage or fibrotic scar tissue (1–3). Although the human endometrium is the only adult tissue that undergoes regular and repeated cycles of destruction and repair under normal physiological conditions (4) parallels may be drawn between mechanisms of post menstrual endometrial repair and the wound healing response of the oral mucosa which also heals without a scar (5–7).

Whilst morphological and cellular changes that occur during the various phases of the human menstrual cycle have been well documented and extensively studied much less is known about the temporal and spatial changes in tissue function that occur during the menstrual phase due to the challenge of timing collection of human tissue during this phase (8). One of the most revealing studies to document the appearance of the endometrium at the time of menses used a combination of a hysteroscopic, histological and scanning electron microscopy. Examination of the surface of the endometrium during initial phases of menses revealed that tissue shedding and repair was not uniform but rather a “piecemeal process” which occurred simultaneously in regions throughout the uterine cavity with the authors suggesting the stromal compartment played an important role (9). One of the most well established mechanisms triggering tissue breakdown is the rapid fall in progesterone which occurs with involution of the corpus luteum in a non-fertile cycle (8). Progesterone also plays a pivotal role in stimulating changes in gene expression and cell function resulting in transformation of the stromal cells so that they secrete factors essential for successful implantation–a process collectively known as decidualization (10). During the normal cycle decidualization is limited to the luminal (functional) layer of the endometrium and this is also the region of tissue shed at menstruation. The occurrence of menstruation is associated with spontaneous decidualization, as opposed to decidualization induced by a fertilization event, and is limited to the higher-order primates including humans, four species of bat, the elephant shrew (11) and a Spiny mouse species (*Acomys cahirinus* (12, 13).

Studies in animal models have included those in primates that spontaneously menstruate such as the baboon (14) as well as species such as the macaque where menstruation can be induced by hormonal manipulation (15, 16). These models have been a valuable complement to studies on human tissue providing an opportunity to harvest samples that include the full thickness of the endometrium at defined timepoints during progesterone withdrawal to explore differences between gene expression in basal and functional zones (17). The classic studies undertaken by Markee (18) used rhesus tissue grafted into the ocular cavity allowing for direct observation of vasoconstriction in the spiral arteries. Notable results from primate studies have included time-dependent expression of metalloproteinases in endometrium (15) (14) and endometrial grafts (19), studies on hypoxia and expression of angiogenic factors such as VEGF (20, 21). Other models have included transplantation of human endometrial tissue into immunocompromised mice and *in vitro* culture of human tissue explants (16).

In addition to the clear intrinsic benefits of improved understanding of the mechanisms that regulate endometrial shedding and repair, this understanding is the basis for development of improved therapies for disorders such as heavy menstrual bleeding and endometriosis (1, 22). The endometrium may also serve as an exemplar of scarless repair with the potential to inform comparative studies and improve our understanding of chronic disease processes such as fibrosis.

## What Are the Advantages and Barriers to Using Mice For Studies on Endometrial Repair?

Whilst the uterus of rodents and women share a common architecture (luminal and glandular epithelial layers, complex stroma, myometrial muscle layers) the common, inbred species of laboratory mice and rats have relatively short “oestrus” cycles with four phases (proestrus, oestrus, met-oetrus, dioestrus) without spontaneous decidualization or cyclical tissue breakdown.

One of the incentives to develop and use mice in studies on endometrial function is the availability of a wide range of genetically modified animals including those using fluorescent protein to identify active promoters, to identify specific cell populations, and targeted deletion of genes either ubiquitously, in a cell-specific manner or following timed induction (23, 24). For example, in *Pdgfrb*-BAC-eGFP mice GFP is expressed under the control of the PDGFRbeta promoter (25) and a recent study has shown that the GFP is expressed in the cells of mesenchymal origin in the mouse endometrium mirroring the expression of the endogenous protein (26). Single cell gene expression analysis of GFP+ cells recovered from cycling endometrium of *Pdgfrb*-BAC-eGFP mice has identified five different populations of cells in the stroma including three transcriptionally distinct populations of fibroblast (26). Targeted deletion of the estrogen receptor alpha gene (*Esr1*) has been a powerful technique which when applied to studies on the mouse endometrium has provided novel insights into the importance of the stromal compartment in estrogen receptor dependent control of epithelial cell proliferation (27). Likewise our understanding of the pivotal role of progesterone in decidualization, fertility and regulation of downstream genes including those of the Wnt pathway has been illuminated by genetic manipulations involving the progesterone receptor gene (28).

## Development and Refinement of Mouse Models of Endometrial Repair (Menstruation)

To overcome the critical limitation that mice lack a spontaneous decidualization response and provide a platform for improved understanding of the mechanisms regulating human menstruation mouse models based on hormonal manipulation have been developed: the most widely used involves ovariectomy of mice and was first reported by Finn and Pope in the 1980's (29), the second relies on induction of pseudopregnancy (30).

### Ovariectomy Model of Endometrial Repair

Briefly, adult female mice are ovariectomised, allowed to recover for 7 days to deplete endogenous hormones, primed with a hormone schedule to mimic the fluctuating hormones experienced by women during the menstrual cycle (estrogen priming and progesterone administration) and the endometrium artificially stimulated to induce decidualization, a process normally initiated following the arrival of a blastocyst in this species. A number of variations on this model have been reported but in all cases tissue breakdown was, as in women, triggered by cessation of progesterone stimulation (simulating CL demise).

In the original model reported in the 1980s 7 days after ovariectomy mice received daily injections of oestradiol (E2, 100 ng/100 μl, 2 days); 3 days of rest, 3 days of injections of E2 (20 ng/100 μl) and progesterone (1 mg/100 μl) followed 4–6 h later by exposure of the uterus and intraluminal injection of peanut oil (29, 31). One problem with this model was variation in the extent of decidualization, however when it did occur removal of progesterone resulted in tissue breakdown accompanied by tissue necrosis, inflammation and luminal shedding (29). Notably the authors recorded changes in the stromal compartment which started with the congestion of dilated blood vessels followed by breakdown of the vessel walls and extravasation of blood. The basal area (outer ring) of the stroma proximal to the myometrium did not take part in the degenerative process but a central core of blood cells and degenerating decidual cells became detached and was shed into the lumen (29). Animals treated in exactly the same way but with the omission of the decidual stimulus did not show such changes in the stroma consistent with data from menstruating species which highlight the importance of stromal cell differentiation as a pre-requisite for the process of menstrual shedding (10).

The model was updated by the Salamonsen Group who modified the protocol to use inbred mice and to include a silastic progesterone-secreting pellet to replace progesterone injections thus ensuring a steadily increasing concentration of circulating progesterone, considered to be more comparable to what happens in women (32). Using this model decidualization was successfully induced in the uterine horns and endometrial breakdown was initiated 12–16 h following progesterone withdrawal. The entire decidua was detached and shed at 24 h and re-epithelialisation of the luminal surface was almost complete by 36 h. Notably this study was the first to define the endometrial breakdown and repair phase as being complete 48 h following withdrawal of progesterone (32). Wang and colleagues (33) investigated the critical time window for progesterone withdrawal using the Salmonsen group model with induction of decidualization by injection of acarchis oil into the lumen of one uterine horn on day 9 and removal of the pellet 49 h later. They reported that replacement of progesterone at 8 and 12 h after pellet removal blocked menstrual-like bleeding while replacement at 16–24 h had no effect and tissue shedding still occurred (33).

**Figure 1:**
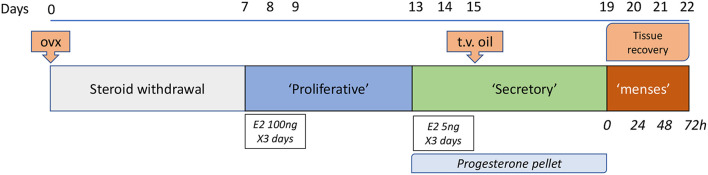
Edinburgh Mouse model of menstruation. This model is based on the pioneering work of Finn and Pope (29) with several refinements including the use of a progesterone pellet (32) and induction of decidualization via a trans-vaginal [t.v.; (3)] route. Samples are recovered on day of pellet removal (time 0, full decidualization) and at times thereafter between 4 and 72 h. Twenty four hours after pellet withdrawal has been characterized as a time of maximal tissue breakdown but by 48 h the epithelial layer surrounding the uterine lumen is typically fully restored.

A further refinement to this model was reported by researchers in Edinburgh (Figure 1): specific changes included induction of decidualization by transvaginal injection of sesame oil directly into the uterine cavity thereby avoiding an additional abdominal surgery as well as an increase in the duration of progesterone administration (pellet in place) from 2 to 4 days after oil exposure ensuring a more robust and reproducible decidualization response (3, 34). In common with other reports shedding was maximal at 24 h after progesterone withdrawal, the luminal epithelial layer was typically intact at 48 h and tissue architecture resembled control intact mice by 72 h. Overt vaginal bleeding was recorded.

Menning and colleagues also reported overt bleeding following induction of decidualization by injection of sesame oil into the uterus followed by removal of a P4 implant 4 days later (35): they recorded time dependent bleeding which was maximal at 24 h and used a tampon collection system to quantify the amount of blood providing a platform for testing drugs including those that modulate angiogenesis (35).

Peterse et al. (36) conducted a study to see if they could maximize the amount of decidual tissue that could be generated for use in a syngenetic model of endometriosis (37) they compared the response when the intrauterine oil stimulus was delivered via the vagina or via surgical laparotomy with the idea that the latter might be more “traumatic” to the tissue and therefore potentially elicit a more robust response (36). Decidualization was achieved in more than 83% of mice with significantly higher rates of bicornate decidualization in the laparotomic group (89%) compared with vaginal administration (38%) suggesting the former was useful if the priority of the study was to maximize decidual material but at the cost of extra surgical intervention.

### Pseudopregnancy Model

Pseudopregnancy can be achieved by mating intact female mice to vasectomised males combined with artificial induction of decidualization. For example, Fan et al. mated adult female CD-1 mice with vasectomized males of the same strain combined with direct injection of 20 μl sesame oil into each uterine lumen on day 4 of pseudopregnancy. On day 6 (2 days after oil injection), bilateral ovariectomy was performed to remove steroids and induce endometrial breakdown (30). When this pseudopregnancy model was used in combination with inhibition of Wnt7a endometrial repair was not normal with a failure of re-epithelialisation and degradation of the basal gland noted (38). In a modified version of the pseudopregnancy mouse model Rudolph *et al* administered the potent progesterone receptor antagonist mifepristone 2 days after induced decidualization instead of performing ovariectomy. This blockade mimicked progesterone withdrawal and stimulated decidual tissue breakdown. Bleeding was evaluated by vaginal lavage and was also visible at the opening of the vagina (39). Recently Wang et al used a pseudopregnancy model to investigate the impact of stress signals on menstrual breakdown (40) reporting that acute stress resulted in an increase in corticosterone contributing to more rapid breakdown and shedding of the endometrium.

In general the ovariectomy model is more widely used as it is very well established and usually considered to provide a more reliable and reproducible timeframe for endometrial breakdown and repair.

## Mechanisms Implicated in Endometrial Repair Identified in Mouse Models

### Hormonal Regulation

In women the menstrual phase of the cycle is characterized by low circulating concentrations of ovarian derived oestrogens and progesterone suggesting that endometrial repair processes are steroid independent. This question has also been addressed in the mouse models of menstruation. The standard ovx+ mouse model of menstruation (Figure 1) is characterized by depletion of ovarian hormones with both shedding and repair occurring in the absence of endogenous oestrogens (3, 8). Kaitu'u-Lino and colleagues argued that other sources of oestrogens, including phytoestrogens in the diet and local metabolism in fat, might be available and the model could not be considered completely steroid-depleted (41). They therefore conducted the model using mice maintained on a soya-free diet and complementing this with administration of aromatase inhibitor letrozole (41). Importantly, no significant difference in the rate of endometrial repair was observed in the complete absence of estrogen, suggesting that this steroid was not essential for complete endometrial restoration in their model.

The presence of abundant androgen receptors in the stromal cells of the basal compartment, which remains intact during menses (42), and evidence for intracrine generation of bioactive androgens within endometrial tissue in response to decidualization (43) led Cousins et al to hypothesize that androgens could modulate the repair process even if the concentrations in blood were low (44). They administered a single injection of the potent bioactive, non-aromatizable androgen, dihydrotestosterone, in parallel with removal of the progesterone pellet. They reported that this treatment increased the duration of vaginal bleeding and delayed restoration of the luminal epithelium with striking spatial and temporal impacts on immunoexpression of MMPs 3 and 9 (44). These results may partially explain why women who have high androgen levels as a result of polycystic ovarian disease sometimes report heavy or extended bleeding during menses (45). Further investigation is required to pin down the precise role of androgens in the endometrial repair process.

### Hypoxia and Angiogensis

Studies on human tissues and in primates have highlighted a role for hypoxia in regulation of endometrial repair processes and angiogenesis (46, 47). Withdrawal of progesterone is associated with an a marked increase in the synthesis of prostaglandins, increased arteriole vasoconstriction and a reduction in oxygen tension within the tissue (47). A key factor in sensing of oxygen tension in tissue is the transcription factor HIF1a (hypoxia inducible factor one alpha) (48, 49). Stabilization of HIF1α in human endometrial tissue has been detected during the secretory and menstrual phase and implicated in regulation of expression of genes involved in angiogenesis including IL8 (46, 50). In an *in vitro* model using human endometrial biopsies it has been shown that P4 withdrawal increased IL8 secretion but only in the presence of hypoxia (50). Coudyzer et al. published contrasting data from a xenograft model where fragments of human endometrium were engrafted to ovariectomised immunodeficient mice: in this model they could not detect evidence for increased HIF1α and concluded that hypoxia is not required to trigger menstrual-like tissue breakdown or repair in human endometrium (51).

The results from studies in the mouse models of menstruation have demonstrated that hypoxia occurs following progesterone withdrawal and that this is also associated with levels of HIF1a and changes in expression of angiogenic genes. For example, Cousins et al. used hypoxyprobe^TM^ to detect low oxygen levels and demonstrated dynamic changes in staining that were consistent with a striking increase in hypoxic conditions during the repair phase and time dependent changes in expression of angiogenesis-associated mRNAs encoded by *Vegfa, Cxcl12, Flt1*, and *Kdr* (34). These results have been complemented by investigations into the role of HIF which can have a dramatic impact on gene expression in low oxygen tissue environments (52). Notably using genetic targeting of *Hif* and pharmacological intervention in combination with the Edinburgh mouse model of endometrial repair Maybin and collaborators were able to manipulate the duration of endometrial shedding simulating heavy menstrual bleeding in women (52) with data supporting manipulation of HIF as a therapeutic target for this prevalent disorder.

The importance of angiogenesis was also confirmed by Menning et al (35) who administered Cediranib, a potent VEGF receptor signaling inhibitor, to mice on days 8 to 15 of their protocol (from day of decidualization to pellet removal) showing a drastic 85% reduction in menstrual like bleeding in treated animals compared with controls.

### Inflammation

The human endometrium hosts a diverse population of immune cells, the abundance and composition of which changes throughout the menstrual cycle. Menstruation has been classified as an inflammatory event because the mechanisms and cellular changes involved are similar to those observed during other physiological inflammatory responses including the increase in the expression of prostaglandins, cytokines and chemokines which are secreted by the decidual cells in response to progesterone withdrawal (50, 53–55). The production of these factors is believed to stimulate the influx of inflammatory cells such as neutrophils and macrophage/monocyte populations (56, 57). Notably induction of excess inflammation in model systems has been shown to be associated with dysregulated repair and fibrosis (58) and may underlie some endometrial pathologies including heavy menstrual bleeding (8). Studies using the mouse models of menstruation have facilitated detailed time-dependent and spatial analysis of the inflammatory process and how it relates to both initiation and resolution of the endometrial menstrual “wound” with some of them highlighted below.

**Figure 2:**
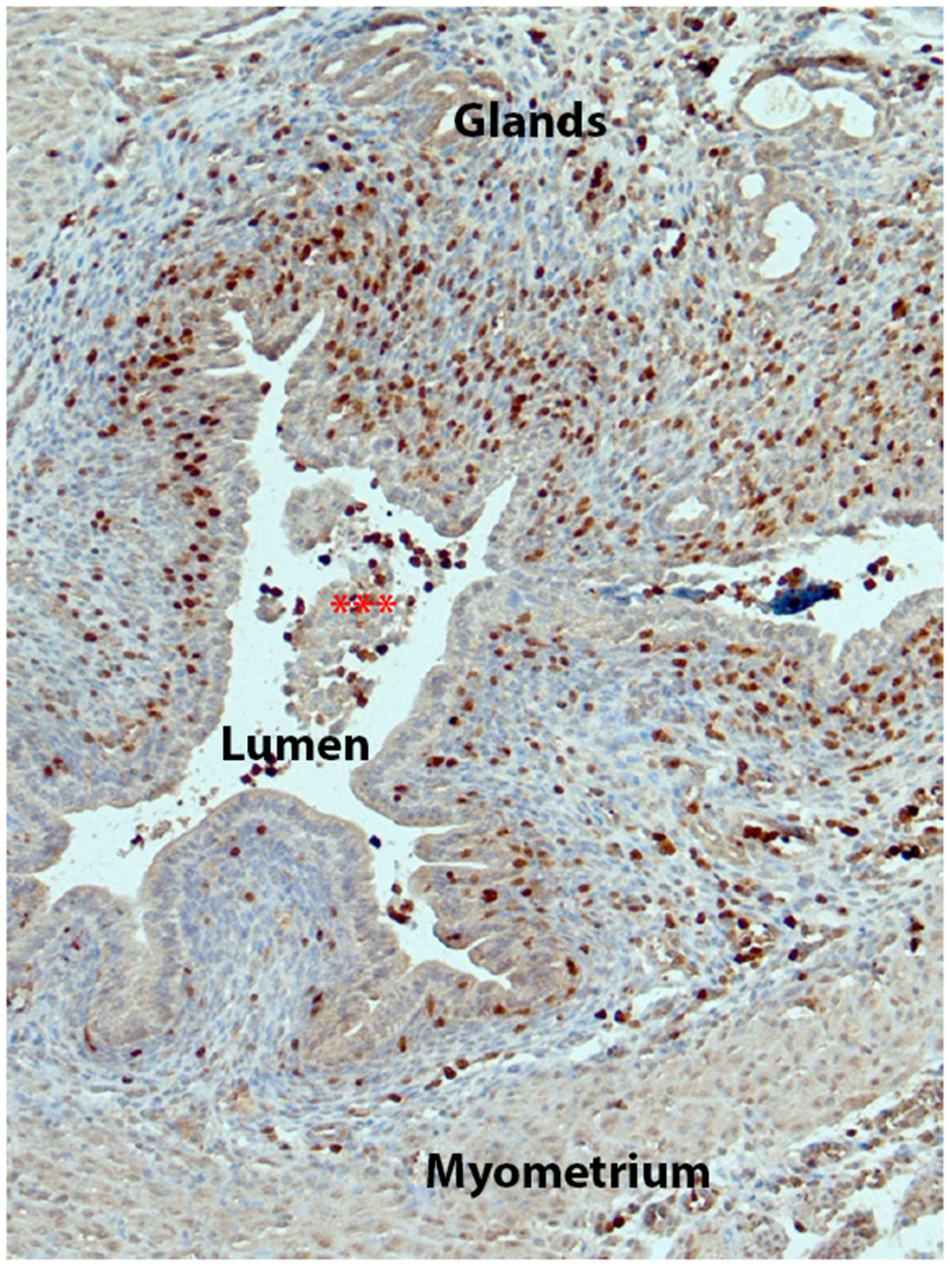
Immune cells of the monocyte/macrophage lineage increase in the mouse endometrium during tissue breakdown. Figure shows endometrium from a Macgreen mouse 24 h after progesterone withdrawal with immune cells identified by immunostaining of GFP (brown, fluorescent images of similar tissues are shown in (23)). Note that there are abundant GFP+ cells in the stromal compartment with many adjacent to the newly intact luminal epithelium.

In the 1980's Finn and Pope reported that one of the first changes in the decidualized mouse endometrium following cessation of progesterone was infiltration of leukocytes into the stroma (31). Subsequent studies have used a wide variety of methods to study the inflammatory response including immunohistochemistry and flow cytometry (3, 35, 56), GFP-labeling of monocyte lineages (23) as well as antibody-dependent cell depletion (59). For example, in their 2003 paper Brasted et al (32) used an antibody directed against CD45 (leukocyte common antigen) to interrogate uterine tissue recovered 0, 12, 16, 20, 24, 36 and 48 h after removal of the progesterone pellet (P withdrawal). Their analysis identified leukocytes in decidualized tissue often in close association with the luminal epithelium, throughout the basal zone and close to the newly regenerated epithelium at later time points. Notably they identified some of these cells as macrophages based on their morphology (32). Manning and colleagues used flow cytometry to analyse tissue digests recovered at 0, 24 and 72 h time points. They reported a massive increase in of CD45+ cells so that they comprised ~10% of the decidua at time zero (mostly NK cells, macrophages and granulocytes) with a striking increase in granulocytes (Gr1+/F480) making up 90% of immune cells during maximal tissue shedding (24 h). Armstrong et al compared tissue sections from human and mouse stained with antibodies directed against neutrophil elastase or GR1 respectively to focus on the neutrophil subtype of granulocytes demonstrating they increased at 8 hours after progesterone withdrawal and at 24 h (56) mimicking results in women and in agreement with other data from ovx models (23, 35, 56). Cousins et al used transgenic “Macgreen” mice in which enhanced green fluorescent protein (EGFP) is expressed under the control of the c-fms promoter (encodes CSF-1R) expressed in the monocyte phagocytic lineage in the mouse (60) as well as some neutrophilic granulocytes (60). Using this lineage marker they were able to shed new light on the dynamic changes in monocyte derived immune cells over the course of tissue breakdown and repair [Figure 2 (23)]. One of the main findings from their study was that distinct populations of “classical” monocytes (GFP+F4/80–), monocyte-derived macrophages (GFP+F4/80+) and a population of putative tissue-resident macrophages (GFP–F4/80+) that became localized to different regions within the tissue during breakdown, repair and remodeling suggesting cells of the monocyte lineage may play distinct roles in these processes (23). The recent application of single cell sequencing analysis of human endometrial tissue is likely to complement these findings by identifying immune cell subpopulations although datasets have not had sufficient depth of read to enable this (61).

The role(s) of immune cell populations have also been investigated using antibody depletion. For example, using the anti-mouse GR-1 antibody Menning and colleagues reported that cells positive for this marker (assumed to be neutrophils) played a role in regulating the expression of matrix modifying enzymes such as MMP3, 9 and 10 and their depletion impaired tissue repair (35). In another study cells were depleted using the anti-GR1 (clone RB6-8C5) antibody and a delay in endometrial repair reported that was concluded to be a consequence of neutrophil depletion (59). The anti-GR1 (clone RB6-8C5) antibody binds to both Ly6G expressed solely on neutrophils and Ly6C expressed on neutrophils, monocytes and subsets of CD8 T cells (62) and therefore cannot be considered to be specific to one of these cell types. Notably Cousins et al (23) used the same RB6-8C5 clone in their studies and reported that many of the GFP+(Ly6G-) monocytes they detected were also GR1+ hence depletion with this antibody is likely to target cells in addition to neutrophils and the immunostaining performed by Armstrong may need to be reevaluated (56).

In women there are well-documented increases in inflammatory chemokines and cytokines that coincide with the withdrawal of progesterone including CCL2 (MCP-1), CXCL8 (IL-8), IL-6, TNF, and COX-2 all showing increased expression in the late secretory and menstrual phases of the menstrual cycle (reviewed in (8)). Complementary studies in the mouse models have extended these findings. For example, Menning et al highlighted the very rapid and transient increase in expression of *Cxcl2, Ccl3, Tnf*, *Il6* and *Ccl2* (35). Other studies reported similar findings for *Ccl2, Il6* and *Cxcl8* (56).

Increased prostaglandin biosynthesis is also an important regulator of inflammatory processes during menstruation (8) that has been explored in the mouse “menstrual” models. In mice as in women induction of a menstrual like event is associated with increased expression of COX-2, an inducible enzyme that acts as a key regulator of the biosynthesis of prostaglandins from arachidonic acid (35, 63). Xu and colleagues used the mouse ovx model to demonstrate that administration of either a nonspecific COX inhibitor (indomethacin) or the COX-2 selective inhibitor DuP-697 led to less influx of leukocytes and inhibition of the menstrual-like process (63).

### Tissue Breakdown

Studies in human tissue have highlighted a critical role for enzymes including matrix metalloproteinases in the destruction of the extracellular matrix (ECM) which is an essential step in tissue breakdown and shedding (64, 65). Historically, elegant studies in rhesus monkeys demonstrated a rapid rise in MMPs (stromelysins/matrilysin; MMP7, MMP3, MMP10) in the luminal portion of the endometrium in response to progesterone withdrawal (19).

Using their mouse model Kaitu'u-Lino *et al* examined the distribution of MMPs revealing an important role for MMP7 and MMP9 during endometrial tissue breakdown, and MMP3 and MMP7 during re-epithelialisation (66). However treatment of mice with the MMP inhibitors doxycycline and batimistat, both of which effectively reduced MMP activity, did not appear to have significant effects on endometrial breakdown or repair (66). The mouse model has been further used to demonstrate dynamic expression and functional importance of ECM interactions (67) and the expression of activin A in specific epithelial and stromal cell populations which may have a role in regulating re-epithelialisation (68). In their 2012 paper Menning et al documented dynamic time-dependent changes in mRNAs encoding Mmp1, 2, 3, 7, 9, 10, and 11. Cousins et al identified changes in the spatial and temporal expression of both MMP9 and MMP3 during the breakdown and repair phases (3, 12) which appeared consistent with the influx of immune cells known to produce MMPs highlighting the ability of the models to recapitulate changes seen in human tissue.

### Epithelial Migration and Proliferation

Kaitu'u-Lino *et al* also used the mouse model to explore the role(s) of epithelial proliferation and progenitor cells in endometrial repair (69, 70). In one study newborn mouse pups were pulse-labeled with bromodeoxyuridine (BrdU) and chased for 5 week before decidualization, endometrial breakdown, and repair were induced (70). In the second study adult mice were also pulse labeled with BrdU immediately after induction of the same model. They reported that very rapid dilution of bromodeoxyuridine label was observed in the luminal epithelium consistent with rapid proliferation, whereas label within the glandular epithelium remained constant. In contrast during the later repair phase glandular epithelial cells had a decrease in detectable BrdU. The authors concluded that a population of epithelial progenitor cells may reside in the basal glands and contribute to postmenstrual repair (69).

In the studies by Cousins et al. they also reported rapid proliferation of epithelial cells including those remaining at the un-denuded surface of the luminal epithelium as well as some stromal cells and epithelial cells surrounding glands (3). In the conclusion of their paper they suggested that re-epithelialisation involves epithelial cell proliferation, epithelial cell migration and transformation of a subpopulation of stromal cells into those with epithelial characteristics in areas where the surface was denuded of epithelial cells (3). These studies provide new ideas about the mechanisms that might operate in parallel to ensure rapid repair of the luminal epithelial cell layer but require further interrogation and testing.

### Mesenchyme to Epithelial Transition (MET)

One of the most striking features of endometrial shedding in women is the piecemeal loss of epithelium resulting in areas of denuded stroma (9). In mice the shedding of the decidual mass is not as piecemeal but it does result in areas of denuded stroma and it was in this region of the endometrium that Cousins and colleagues detected stromal cells which co-expressed vimentin and cytokeratin during the most active phase of endometrial repair (24 h after progesterone withdrawal) (3). These authors also analyzed the expression of genes implicated in mesenchymal-to-epithelial transition across the time course of the repair process with evidence of changes in expression of regulatory genes including *Wt1* and members of the snail/slug family that are known to play a role in regulation of MET (3). Whether this is a transient change in the stromal population or is part of their differentiation into a functioning epithelium required further investigation.

Studies on postpartum endometrial repair have also provided evidence that MET occurs (71, 72). Specifically, the authors used genetic manipulation to allow for fate mapping of uterine cells expressing *Amhr2* using beta-galactosidase (71) or EGFP (72). In both studies positive signal (blue/EGFP) was restricted to stromal cells and myometrium in normal cycling mice but following parturition when there is extensive damage to the endometrial tissue, some of the labeled cells transformed into cells with epithelial characteristics, including expression of cytokeratin, and became incorporated into the luminal and glandular epithelial cell layers (71). In their 2013 study Patterson *et al* also used the mice in combination with the pseudopregnancy menses model described above and reported co-localization of vimentin (stromal marker) and cytokeratin (epithelial marker) in cells within the basal zone close to the myometrial border that peaked at 48 h post-ovx (72). Despite the location of these putative MET cells being different to that reported by Cousins (3) likely reflecting differences between the two models, these data further support a role for MET in post-menstrual repair.

A recent paper by Ghosh et al. (73) challenged the idea that MET was involved in maintenance and regeneration of the epithelium of the endometrium and oviduct. Specifically, they conducted a comprehensive examination of embryonic and adult reproductive tracts using LacZ reporter lines driven by promoters for *Amhr2, Sm22, Cspg4, Thy1*, and *Pdgfrß* to explore whether epithelial cells expressing reporter protein arose in adulthood from MET or had an embryonic origin because they were induced at a time when cells had meso-epithelial characteristics. In all cases they attributed epithelial expression of the reporter protein in adulthood to activation of the promoters during embryonic life ruling out MET in adult cycling mice (73).

Some of the findings summarized above are consistent with endometrial stromal cells having an inherent “plasticity” to change their phenotype from that of mesenchyme to one more consistent with epithelium. In addition to the studies on the menstrual models it is notable that decidualization might be considered as a form of hormone-induced MET with endometrial stromal fibroblasts acquiring epithelioid characteristics, such as expanded cytoplasm, rough endoplasmic reticulum, and a reorganized actin cytoskeleton (30). We postulate that this feature of endometrial mesenchymal cells may be an important contributor to the resilience of the endometrium to acute insults such as the breakdown and shedding of endometrium at the end of each menstrual cycle but further studies including those using lineage tracing are required to confirm this.

### Progenitor/Stem Cells

Cells with stem cell-like properties, such as high proliferative potential, multilineage differentiation ability *in vitro* (adipo-, osteo- and myo-genic), and expression of stem cell-associated markers, have been identified in the human endometrium [basal compartment, perivascular location, PDGFRβ+CD146+, SUSD2+; (74, 75)], but the precise contribution of these cells to cyclical endometrial repair mechanisms remains the subject of intense investigation. Recent progress has included use of specific surface markers for isolation of progenitor/stem populations from tissue samples and menstrual effluent with novel applications proposed for regenerative medicine and tissue repair (76, 77). The role of stem/progenitor cells has also been investigated in the mouse model of menstruation although this has been challenging due to the lack of a specific lineage marker. A study by Kaitu'u-Lino *et al* using the LRC technique reported results suggesting that a population of epithelial progenitor cells might reside in the basal glands and that stromal LRC, located in a perivascular location could have an active role to play in endometrial repair (70). Despite evidence for the presence of multiple lineage-restricted stem/progenitor cell populations within the human/mouse uterus, the exact contribution to endometrial tissue repair remains elusive in part due to a lack of definitive markers. A recent study by Kirkwood et al identified an equivalent population of perivascular PDGFRβ+CD146+ cells in the mouse endometrium and demonstrated exclusive expression of NG2 (Cspg4) (26). The emergence of such novel identification markers will allow for their specific role in endometrial repair and regeneration to be interrogated.

## Can We Translate Knowledge Gained From Studies on Endometrial Repair to Treat Endometrial Fibrosis?

Endometrium repair is not always scar-free and intrauterine adhesions can occur as a result of a fibrotic response within the basal layer and is associated with poor pregnancy outcomes (78). The existence of these intrauterine adhesions is usually referred to as “Asherman's syndrome” (AS) with risk reported to be increased by repeated miscarriage, cesarean section and surgical removal of uterine contents [curatage; (79)]. Mouse models of AS have been developed by inducing a fibrotic response within the uterus by repeated “wounding” with a needle (80, 81). These models have been used to the test the ability of cell-based therapies to improve fertility, the rationale being that stem/progenitor cells may be involved in endometrial regeneration (82) and have been successfully applied for tissue repair in models of prolapse (77). One paper reported the use of human perivascular stem cells (hPVSCs) from umbilical cords was able to rescue the poor pregnancy outcome in AS mice via HIF1-dependent angiogenesis (83). Other studies have used mesenchyme cells derived from cultured human pluripotent stem cells (81) or from bone-marrow derived stem cells also with some promising results (84). A recent review considered a wider range of different sources of mesenchyme stem/stromal cells including menstrual blood [as discussed above, (76)] as well as evidence that extracellular vesicles secreted by these cells might also be considered as a cell free therapy for AS (85) which, given the logistical challenges of cell therapy, deserves further investigation.

Recently the importance of inflammatory pathways in the etiology of AS has gained more prominence (86) and this would be in agreement with their central role in endometrial repair (discussed above) as well as in the development of fibrosis in other tissues such as the liver (87). In a recent study immunostaining of endometrial tissue from 10 patients with AS identified not only increased amounts of fibrosis within the stromal compartment (collagen fibers and smooth muscle actin) but also alterations in macrophage phenotype (88). Changes in macrophage phenotype and pro-fibrotic cell changes are have also been identified in a mouse model of endometriosis (89), and in both disorders there appears to be potential for targeting macrophage phenotype/function as a novel therapy. Further insights from the mouse models of menstruation and comparison to those of AS may help refine the type(s) of immune and cell based therapies that can treat patients and improve their fertility.

## Comparisons Between Mechanisms of Tissue Repair in the Endometrium, Fetal Skin and Oral Mucosa

Unchecked inflammation, fibrosis and scaring in response to tissue injury can result in significant tissue damage and associated morbidity (90). A number of studies have contributed to a greater understanding of the plasticity and heterogeneity of fibroblasts and their role in fibrosis (90). Whilst to date there has been little cross-over between studies using single cell analysis methods to explore fibroblast heterogeneity in fibrosis-prone tissues (90) and those using similar methods to interrogate endometrium in human (91) or mouse (26) this is clearly a topic that could be explored using existing data and bioinformatics to see if any of the endometrial cell subtypes have unique gene signatures. As the new single cell datasets have only recently been generated to date most attention has been paid to considering mechanisms that might explain scar-free healing of skin in the fetus and (92, 93) and lining of the mouth (94, 95) with a strong focus on exploring mechanisms that might be manipulated therapeutically in other sites (96).

A recent review summarized information obtained from studies using mice which have identified significant differences between gene expression in fibroblasts, deposition of extracellular matrix, the numbers of immune cells, expression of inflammatory regulators (IL33, prostaglandins) and metalloproteinases (MMPs) in fetuses where skin repair is rapid and scar-free (E15) and when scars are formed (E18/19) (93). In a detailed study using single cell fate mapping and 3D confocal imaging Jiang and colleagues identified two different fibroblast lineages that are responsible for the transition from scarless to skin scaring, again highlighting the importance of this cell type (92). Consistent with the results reported in fetuses repair of the oral mucosa also heals more rapidly than adult skin. In a recent study using nude mice, fibroblasts from the oral mucosa were shown to improve healing rates of adult skin wounds (97).

The inflammatory component of wound healing in the oral mucosa is associated with lower numbers of immune cells including macrophages, when compared to wounds of equivalent size in the adult skin, as well as decreased expression of the pro-inflammatory cytokines IL-6 and TGFβ1 (98). An animal model that can augment our understanding of skin repair is the African Spiny mice (Acomys) where cutaneous repair in adults closely resembles that of fetal stages of laboratory mice. Notably in this species skin repair is also associated with less inflammation, reduced collagen secretion and reduced numbers of macrophages mirroring findings in fetal mice (99). These results appear at odds with the wound response of the endometrium in which progesterone withdrawal triggers increased expression of inflammatory cytokines as well as a rapid increase in the numbers of immune cells including macrophages (8) but this may reflect the difference in the time scale and tissue response involved with endometrium breaking down and shedding over days whereas studies on skin wounding have focused on acute, usually incisional insults. Further comparisons between the inflammatory responses in skin and endometrium will be useful in finding both similarities and differences.

**Figure 3:**
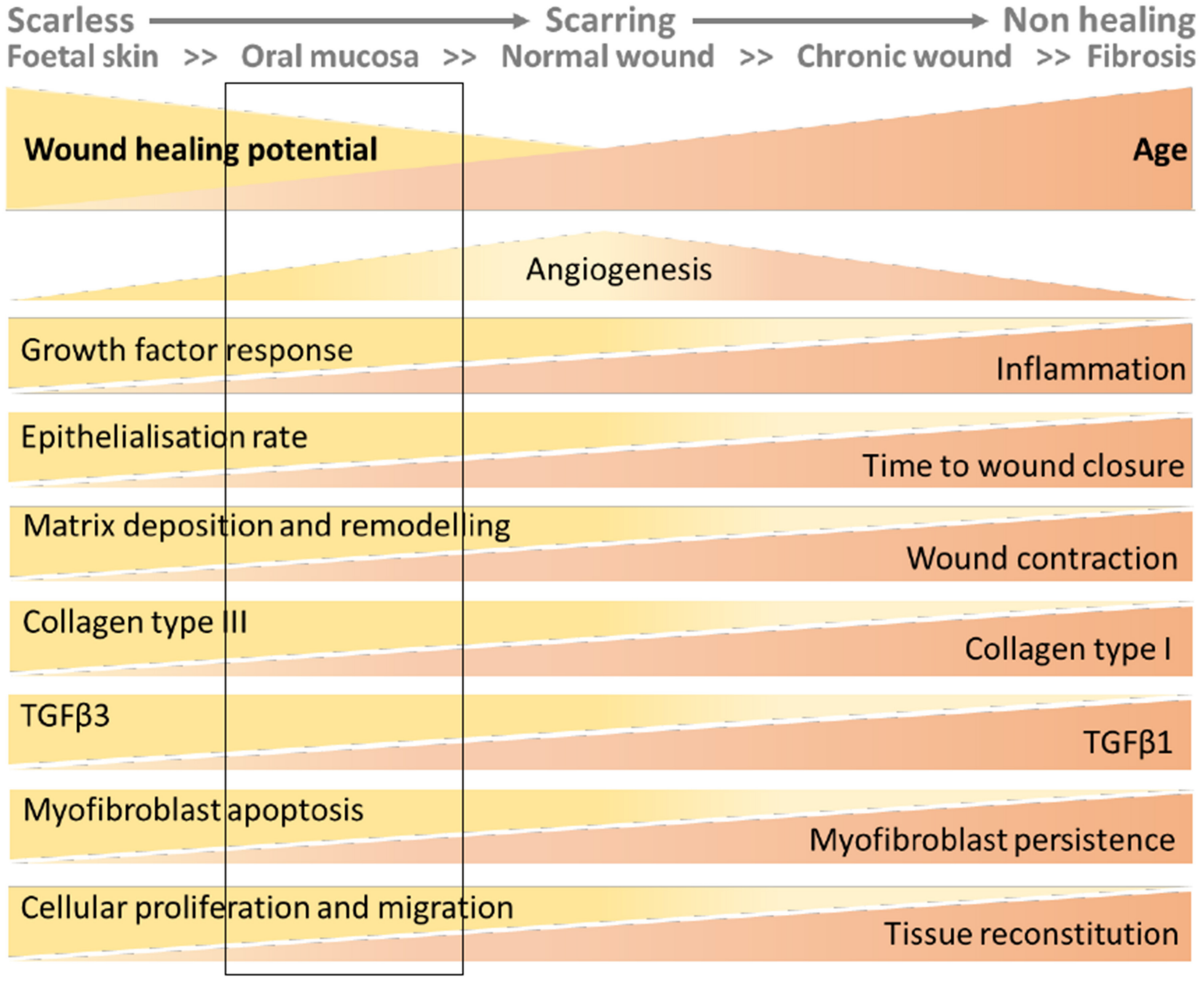
Wound healing continuum with inclusion of putative location of endometrial tissue based on data from the mouse models. The figure has been adapted from that published in (100). The rectangular box added to the figure represents the characteristics of endometrial wound repair based on the interrogation of the mouse models described in this review.

In summary, endometrium, fetal skin and oral mucosa all heal more rapidly than adult skin. Fibroblasts play a key role in regulating the efficiency of the repair processes in all these tissues. If we represent wounding of the skin as a continuum from scar-free in the fetus to the non-resolving wounds associated with aging (100) the endometrium would appear to most closely align with that of oral mucosa with rapid repair but potential for fibrosis (Figure 3).

## Summary and Conclusions

The endometrium is a remarkable tissue which may experience 400 cycles of repeated breakdown, shedding and repair during a woman's lifetime with restoration of tissue architecture so that it is able regenerate and transform into a receptive state to receive the blastocyst during the next menstrual cycle. Endometrial repair is tightly regulated both temporally and spatially and mal-adaptations to the mechanisms responsible result in disorders including heavy menstrual bleeding (inefficient repair?) and Asherman's syndrome (intrauterine fibrosis/excess repair?) (1, 8). Whilst the common laboratory species of mouse do not naturally experience menstrual cycles protocols based on manipulation of hormones, artificial induction of stromal cell decidualization, and acute withdrawal of progesterone have led to the development of robust and reproducible induction of a “menses-like” event in the mouse endometrium. Comparison with human tissue samples shows that these models recapitulate the key physiological changes associated with menstruation. Specifically local/focal hypoxia, spatial and temporal expression of metalloproteinases, increased expression of angiogenic factors and inflammatory mediators, epithelial cell proliferation and the influx of large numbers of immune cells. An intact luminal epithelial layer is rapidly restored and the tissue appears “unwounded” within 48–72 h of progesterone withdrawal. Studies in mice have provided the platform for testing drugs and cell depletion to better inform new therapeutic opportunities for women's health disorders.

It is anticipated that further studies on the mouse models of menstruation, including more extensive comparison to regeneration and repair mechanisms in other tissues will continue to inform both our understanding of the normal physiology of menstruation but also an important platform for development of new therapies to treat conditions such as heavy menstrual bleeding, endometriosis and Asherman's syndrome.

## Author Contributions

All authors listed have made a substantial, direct, and intellectual contribution to the work and approved it for publication.

## Funding

The University of Edinburgh provides funding to cover costs of open access publishing. Research in the author's group on endometrial biology has been supported by MRC MR/N024524/1.

## Conflict of Interest

The authors declare that the research was conducted in the absence of any commercial or financial relationships that could be construed as a potential conflict of interest.

## Publisher's Note

All claims expressed in this article are solely those of the authors and do not necessarily represent those of their affiliated organizations, or those of the publisher, the editors and the reviewers. Any product that may be evaluated in this article, or claim that may be made by its manufacturer, is not guaranteed or endorsed by the publisher.
